# Computed tomography in the assessment of diaphragm dysfunction: current evidence, comparison with ultrasound, and future perspectives

**DOI:** 10.3389/fmed.2026.1980692

**Published:** 2026-10-28

**Authors:** Xin-Yu Yao, Wan-Li Yang, Ran Zhang, Shi-Wei Li

**Affiliations:** Department of Anesthesiology, Suining Central Hospital, Suining, China

**Keywords:** CT, diaphragm, diaphragm dysfunction, diaphragmatic thickness, ultrasound

## Abstract

The diaphragm serves as a crucial respiratory muscle in the human body, contributing 60%–80% of the work of breathing at rest. Diaphragmatic dysfunction (DD) may arise from multiple factors, including prolonged mechanical ventilation, surgical trauma, and inflammatory responses. This dysfunction escalates respiratory effort, increases the incidence of pulmonary complications, and prolongs hospital stays. Even unilateral DD can cause ventilatory abnormalities and impaired gas exchange, complicating weaning processes in ICU patients and raising the incidence of ventilator-associated diaphragmatic dysfunction. Consequently, safeguarding and monitoring diaphragmatic function is essential, making the accurate identification of DD critical. Ultrasound is currently a widely employed tool for evaluating diaphragmatic function; however, clinical practice has uncovered certain limitations inherent to this method. Relevant studies were sourced from publicly accessible databases, such as PubMed and Web of Science, focusing on CT morphological parameters, diagnostic thresholds, and clinical applications in diverse patient populations. Recent findings indicate an increased application of CT in diaphragm assessment, offering complementary structural information to ultrasound. Despite this progress, existing literature lacks a comprehensive review of CT-based evaluations of DD and comparisons with ultrasound, which has led to the underutilization of CT's potential for rapid and accurate assessment of diaphragmatic function and subsequent guidance for diagnosis and treatment. This article aims to summarize the current applications of CT in assessing diaphragmatic function, compare these findings with ultrasound, analyze the potential advantages of CT, and outline future research directions in this area.

## 1 Introduction

The diaphragm, a thin, dome-shaped musculotendinous structure located at the base of the thoracic cavity and the roof of the abdominal cavity, serves as the primary barrier separating the thorax from the abdomen. Posteriorly, it attaches to the upper lumbar vertebrae and intervertebral discs via its muscular crura; anteriorly, it is fixed to the lower sternum and xiphoid process; and laterally, it inserts into the lower six ribs and costal cartilages. Three major hiata are present, corresponding to the vena cava, esophagus, and aorta, respectively (1). Despite being one of the thinnest muscles in the human body, the diaphragm accounts for the majority of the ventilatory workload (2, 3), facilitates airway secretion clearance (4), and contributes to a reduced incidence of pulmonary complications. Compromised diaphragmatic function may lead to unexplained dyspnea, respiratory failure, or cardiac arrest, marking diaphragmatic dysfunction (DD) as a frequently overlooked but critical cause of respiratory impairment.

Previous studies have established that patients undergoing prolonged mechanical ventilation are vulnerable to diaphragmatic atrophy. In critically ill patients, each 10% decrease in diaphragmatic thickness (DT) correlates with a 1.55-fold increase in ICU mortality and a 1.66-fold rise in hospital mortality (5, 6). Diaphragmatic atrophy significantly contributes to difficulties in weaning from mechanical ventilation. Notably, clinical symptoms of DD generally do not emerge until muscle function declines to 30% of its normal value (7), highlighting the importance of early identification to facilitate timely intervention and treatment (8). A range of assessment tools exists for evaluating DD, each with distinct advantages and limitations. Although ultrasound remains the predominant modality in clinical practice, it has certain shortcomings. In recent years, computed tomography (CT) has gained traction for diaphragmatic evaluation, yet a comprehensive review on this topic has not been published.

## 2 Method

This narrative review retrieved literature from PubMed and Web of Science, focusing on studies published within the last 10 years (2016–present). Search terms included “diaphragm,” “diaphragmatic dysfunction,” “CT,” “computed tomography,” and “ultrasound.” Eligible studies reported CT-related morphological parameters, diagnostic thresholds, or clinical outcomes related to DD. Case reports, conference abstracts, and irrelevant review articles were excluded. Titles, abstracts, and full-text articles were screened sequentially to select qualified evidence for inclusion in this review.

## 3 Ultrasound evaluation of diaphragmatic dysfunction

### 3.1 Ultrasound applications in diaphragmatic dysfunction

Ultrasound was first applied to diaphragmatic assessment in 1969, leading to a growing body of research on its use. Presently, ultrasound is the preferred clinical tool for evaluating DD (9, 10). Due to its non-invasiveness and reproducibility, ultrasound has gradually replaced phrenic nerve stimulation, once regarded as the gold standard (11, 12). Common ultrasound modes, including B-mode and M-mode, are widely used (13, 14), and an international expert consensus has standardized probe selection, operational procedures, and diagnostic criteria for DD (15), rendering ultrasound particularly suitable for critically ill patients. Additionally, ultrasound can screen for perioperative DD, as affected patients may face a 75% reduction in forced vital capacity, leading to impaired ventilatory function (16). Early intervention in such cases is crucial to reducing the incidence of postoperative pulmonary complications. Following ultrasound-guided supraclavicular brachial plexus blockade, the incidence of ipsilateral hemidiaphragmatic paralysis ranges from 67% to 80% (17, 18); however, no effective intervention has been firmly established. Thus, employing ultrasound to evaluate diaphragmatic function in surgical patients receiving brachial plexus blocks allows for early identification of the risk for diaphragmatic paralysis, enabling timely interventions, such as conversion to general anesthesia with endotracheal intubation, to prevent intraoperative respiratory distress. Evidence from Spadaro et al. indicates that diaphragmatic excursion (DE) decreases within 24 h post-thoracic surgery, with DE reduction serving as an independent risk factor for pulmonary complications (19). Furthermore, Daniel and colleagues demonstrated a decline in both the diaphragm thickening fraction (DTF) and DE by the third postoperative day. Ultrasound studies have revealed an incidence of postoperative DD in cardiac surgery patients as high as 38%, with 8% experiencing persistent DD (20, 21). In summary, ultrasound plays a crucial role in the early recognition of perioperative DD.

### 3.2 Limitations of ultrasound

Although ultrasound is currently the preferred diagnostic tool for DD, considerable controversy persists regarding its diagnostic parameters and criteria. First, there is disagreement about the affected sites; some studies indicate that DD is predominantly bilateral (20), while others suggest it can be unilateral and associated with specific surgical procedures, such as lobectomy (19). Second, diagnostic thresholds vary widely; for example, the lower limit of normal DE during quiet breathing has been reported as 0.9 cm for females and 1.0 cm for males, while during deep breathing, these values are 3.3 cm and 3.2 cm, respectively (22). These findings contradict criteria proposed by Spadaro et al., who defined DD as DE <1 cm without distinguishing between sexes or respiratory depth. Additionally, another grading system classifies DD based on the percentage reduction in DE from baseline: a reduction >70% indicates complete DD, 25%–70% indicates partial DD, and <25% indicates no DD (23). Third, the selection of diagnostic indices lacks uniformity; commonly utilized parameters include DE (15, 19, 24–29), DT, and DTF (30–36). Furthermore, the accuracy of ultrasound measurements is subject to operator and position dependence, as well as poor image quality, particularly in left-sided assessments and in obese patients (14, 37). In conclusion, no consensus currently exists regarding involved sites, diagnostic thresholds, grading criteria, or core assessment indices for DD. The diagnostic standards demonstrate considerable divergence, with a lack of uniform differentiation by sex or respiratory status, well-established cutoff values, or validated grading systems; consequently, the choice of indices remains inconsistent. This overall low level of standardization significantly hampers precise and consistent clinical evaluation of diaphragmatic function using ultrasound.

## 4 CT evaluation of diaphragmatic dysfunction

### 4.1 CT applications in the diaphragm

As a static imaging modality, CT facilitates a comprehensive evaluation of diaphragmatic morphology and achieves satisfactory three-dimensional (3D) reconstruction (38). This 3D reconstruction reproduces the stereoscopic structure of various diaphragm parts, intuitively displaying morphology and reflecting functional status during imaging, while also revealing the relationship between the diaphragm and adjacent organs, regardless of whether these organs contain air. This capability addresses ultrasound's major limitation, which is susceptibility to interference from gas. Furthermore, CT allows for morphomic analysis (e.g., assessment of subcutaneous fat, trunk muscle area) to predict patient survival and length of hospital stay (39, 40) and is employed to detect organic space-occupying lesions of the diaphragm. It can directly evaluate 3D structure *in vivo*, assessing muscle mass, function, fiber type, and lipid content (41–44). Established as a primary tool for morphological analysis, CT is also effective for quantifying muscle tissue at the lumbar level (45). Jung et al. noted that the diaphragm is more prone to atrophy than other muscles (44), providing a solid experimental basis for further CT-based analysis of the diaphragm. Additionally, standard chest CT, which is widely used in clinical practice, captures the entire diaphragm, allowing for quantification of its 3D morphology (44, 46, 47).

CT offers high specificity and sensitivity in diagnosing diaphragmatic injuries and acquires detailed imaging information. Currently, multi-slice spiral CT is widely utilized, providing lower radiation doses and greater safety than conventional single-slice CT scanning (1). Compared to ultrasound, CT delivers a more comprehensive depiction of the diaphragm (48), exhibiting broader applicability, improved reproducibility, and operator independence. As a skeletal muscle, the diaphragm can be accurately scanned using CT. Stefano Gatti et al. confirmed that CT-measured thickness of the left posterior pillars of the diaphragm moderately correlates with ultrasound measurements (49). However, CT-based studies focusing specifically on the diaphragm remain relatively limited, primarily addressing the crura and diaphragmatic injuries (50). Jung et al. demonstrated that, under magnetic stimulation, diaphragmatic volume correlates with airway pressure, proposing the feasibility of quantitative CT measurement of the diaphragm (44). Subsequently, Gi Dong Lee et al. were the first to calculate DT in critically ill mechanically ventilated (MV) patients (51), discovering that atrophy occurs not only in the crura but also at the level of the celiac trunk. Yue Nan Ni et al. measured the thickness of the right hemidiaphragm dome in the coronal position, finding a progressive decrease in thickness associated with a higher rate of reintubation (51). Aydın Sanli selected DT measurements at the T11–T12 level (52). These findings indicate that DD may impact various diaphragm regions; however, given the diaphragm's irregular shape, further validation is necessary to identify the most reliable thickness measurement site. Warawut Sukkasem and colleagues demonstrated that CT measurements of DT at the abdominal aorta and L1 level are feasible (53), exhibiting good intra- and inter-observer reproducibility (54). Specific measurement locations included the maximum and minimum DT at the origin of the abdominal aorta in the axial plane (Figure 1A) and anterior, middle, and posterior DT at the L1 level (Figure 1B), as well as maximum DT at the origin of the abdominal aorta(Figure 2A) and anterior DT at the L1 level in the coronal plane (Figure 2B). This research identified optimal and reproducible measurement points and planes for DT assessment on CT, further indicating that CT is a reliable tool for measuring DT and providing a foundation for future studies on CT and DD.

**Figure 1:**
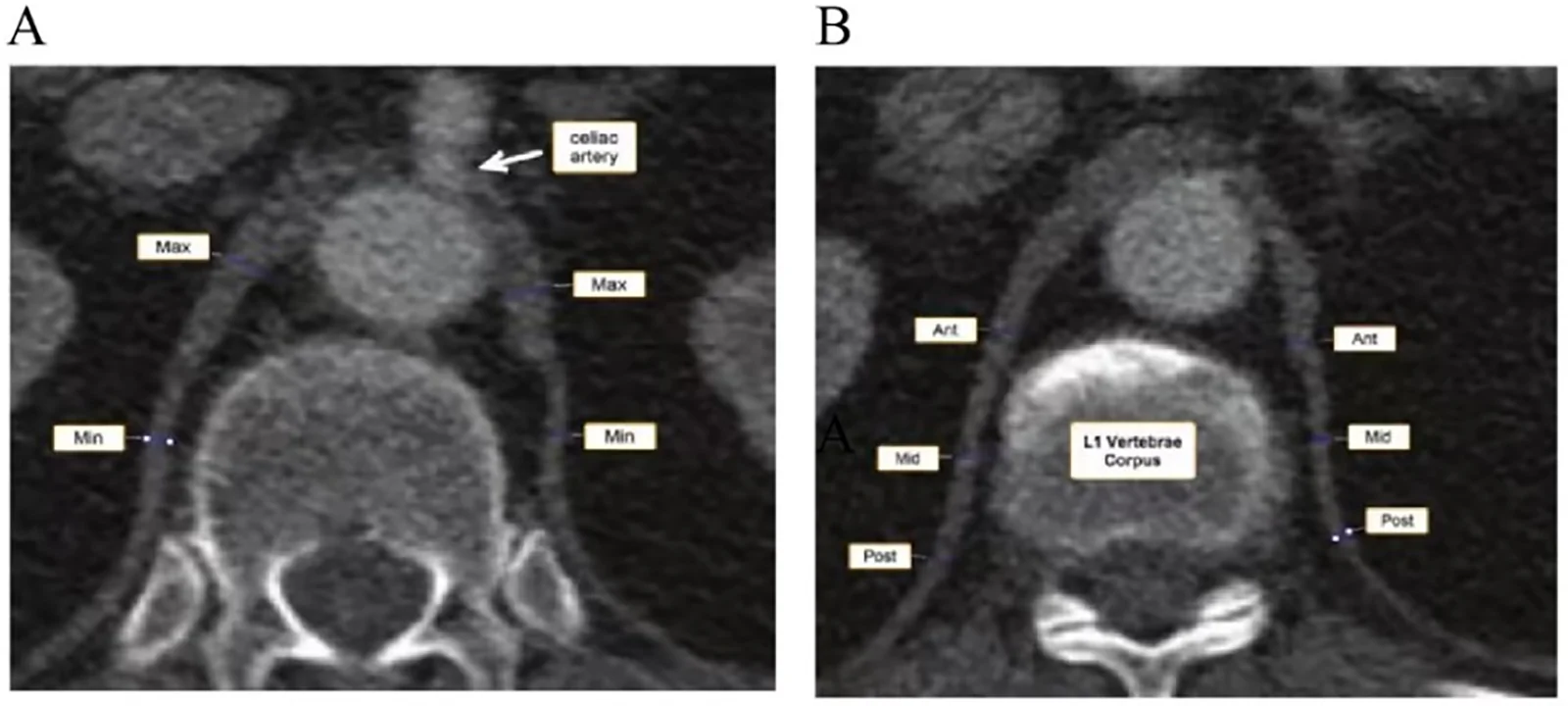
On axial images, diaphragmatic thickness was measured at the abdominal aorta and L1 vertebral levels. The diaphragm manifests as an inverted U-shaped high-density band anterior to the vertebral body. **(A)** Maximum and minimum DT at the origin of the abdominal aorta at the level of the axial plane. **(B)** axial measurements were recorded for each crus at the level of the upper part of the L1 vertebral body along the anterior, mid, and posterior aspects of the vertebral body. (53) 10.1097/RTI.0000000000000276.

**Figure 2:**
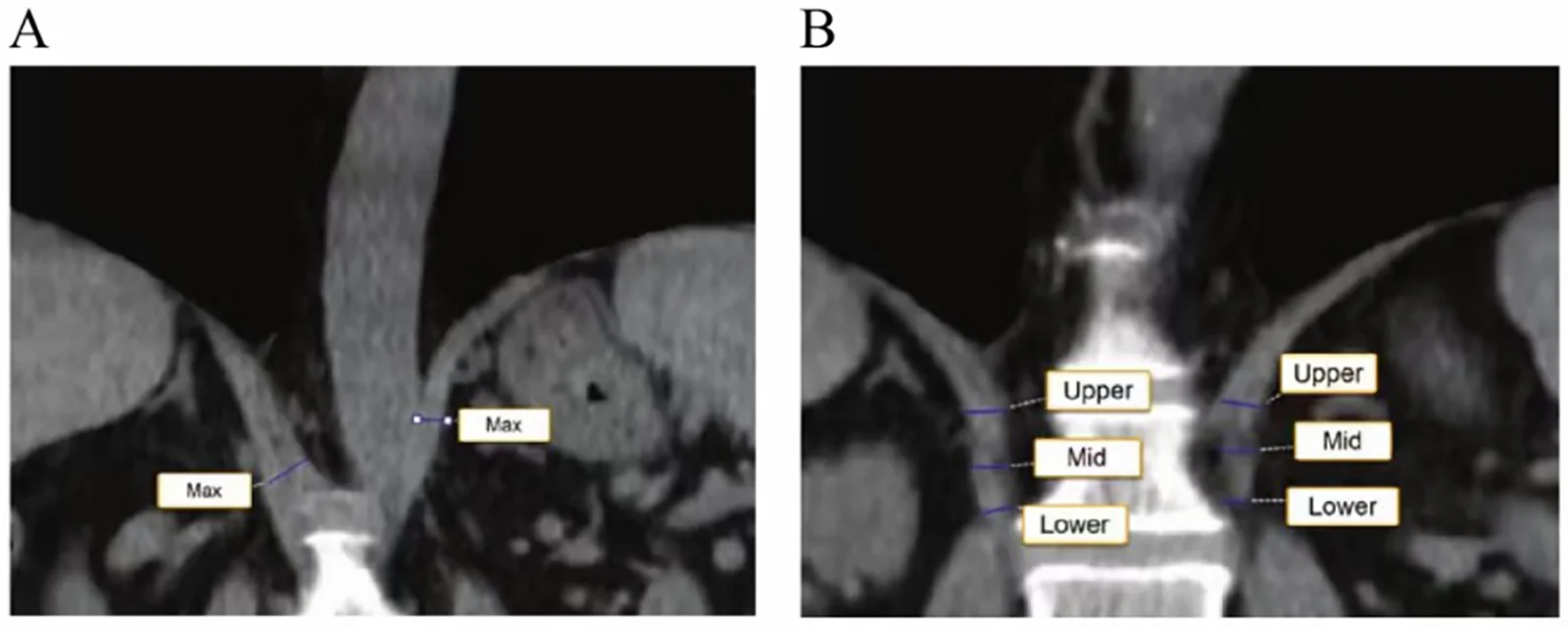
On coronal images, diaphragmatic thickness was measured at the abdominal aorta and L1 vertebral levels. **(A)** Maximum DT at the origin of the abdominal aorta in the coronal plane **(B)** On coronal images, minimal and maximal thicknesses were recorded along the anterior part of the L1 vertebral body at upper, mid, and lower vertebral body levels. (53) 10.1097/RTI.0000000000000276.

### 4.2 CT parameters for diaphragmatic dysfunction

Numerous factors contribute to reduced DT during the perioperative period, including sedation (55), malnutrition (56), oxidative stress (57), systemic inflammation (58), and hypophosphatemia or hypomagnesemia (51). Studies indicate that a decrease in DT is common in humans (59), with an average daily reduction of 6% observed in MV patients (60), which correlates with impaired diaphragmatic function. However, current CT-based research on the diaphragm has primarily concentrated on measuring thickness, while the specific relationship between thickness and function has yet to be explored. As DD is a significant contributor to adverse events in perioperative patients, establishing a link between DT and functional status is essential. In 2024, Pauline Lallement and colleagues utilized CT to correlate DT with other relevant parameters and diaphragmatic function (61). They employed the thickness measurement sites validated by Sukkasem W. and additionally measured the vertical distance from the diaphragmatic dome to the L1 level bilaterally (Figure 3), introducing a novel index. This study confirmed the reliability of DT measurements at the abdominal aorta and L1 levels and determined threshold values of 3.0 mm at the abdominal aorta level and 4.5 mm at the L1 level through ROC analysis, both demonstrating good diagnostic performance (62). These thresholds differ from the 2.5 mm reported by Sukkasem W., likely due to variations in the selected gold standard. Notably, Pauline Lallement's use of ultrasound, being more sensitive, aligns better with current clinical practices as the reference standard. Importantly, the newly proposed index (the vertical distance from the dome to L1) achieved 100% diagnostic accuracy for bilateral DD (62), with thresholds of 4.4 cm on the right and 2.7 cm on the left. This metric, proposed for the first time, may serve as a reliable CT-based parameter for assessing DD. Clinical observations revealed that dome heights on both sides decreased to varying degrees, suggesting that diaphragmatic function may be compromised even in the absence of structural atrophy. Another noteworthy finding indicates that when the excursion of one hemidiaphragm is reduced, the contralateral side may exhibit a compensatory increase, potentially reflecting a neuronal compensatory mechanism (63–65). This phenomenon could lead to false positive identification of DD by ultrasound; however, CT-measured crural thickness showed no significant bilateral changes, providing a new perspective for CT-based assessment of DD and suggesting that more accurate and specific CT indices may be developed in future research. A summary of the relevant CT parameters is provided in Table 1.

**Figure 3:**
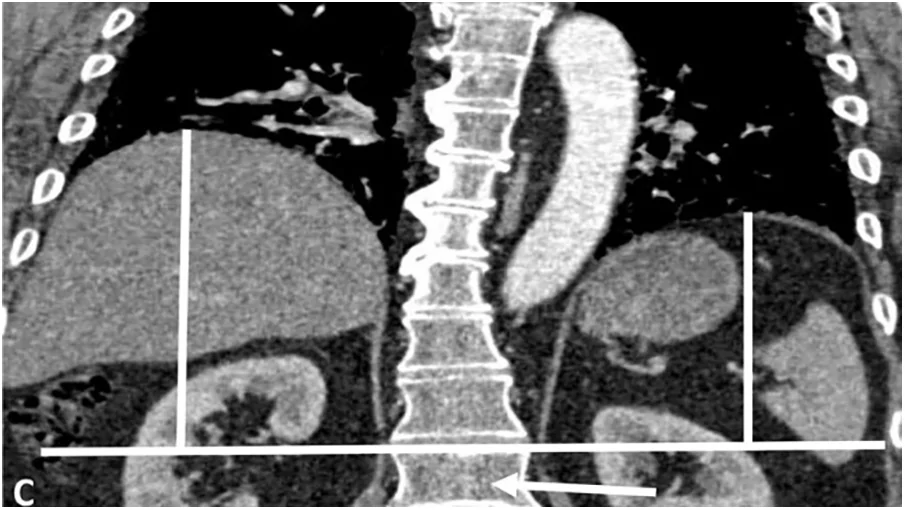
Measurement of the diaphragm height difference. Heights (white vertical lines) from the highest point of the diaphragmatic dome to its perpendicular intersection with a line (white horizontal line) following the upper plateau of L1 (white arrow). (61) 10.1186/s12931-024-02770-w.

**Table 1:** Summary of CT measurement levels and parameters in the diagnosis of diaphragmatic dysfunction.

CT Measurement Level	Parameter	Literature Threshold/Cut-off	Diagnostic Performance	Target Population
Celiac trunk level (origin of abdominal aorta)	DT	3.0 mm (Lallement 2024)、2.5 mm (Sukkasem）	Good ROC performance	Mechanically ventilated and perioperative patients
Anterior/middle/posterior crura at L1 vertebral level	DT	4.5 mm (Lallement)	Distinguishes mild from severe atrophy	Critically ill ICU patients
Vertical distance from diaphragmatic dome (coronal plane) to L1 level	Dome height	Right: 4.4 cm; Left: 2.7 cm	100% accuracy in detecting bilateral DD	Differential diagnosis of unilateral diaphragmatic paralysis
CT density value of the diaphragm	Muscle fatty infiltration index	Lower density indicates more severe atrophy	Predicts severity of COPD	Patients with chronic respiratory diseases

### 4.3 CT evaluation of the diaphragm in patients with chronic respiratory diseases

#### 4.3.1 CT evaluation of the diaphragm in patients with chronic obstructive pulmonary disease (COPD)

Given the availability and feasibility of CT, current studies have demonstrated a close relationship between diaphragmatic function and the respiratory system. Recently, CT has gained traction in the evaluation of respiratory diseases. DD represents a significant comorbidity of COPD and can predict adverse outcomes, including dyspnea and mortality (66). In reconstructing diaphragmatic morphology in COPD patients, reductions in both the length and surface area of the diaphragm were noted compared to healthy controls (67). Donovan et al. also observed decreases in both dome height and CT density of the diaphragm to varying degrees (Figure 4), correlating these changes with COPD severity (68). This study was the first to introduce the concept of diaphragmatic density as an emerging index of “muscle quality” (43). Subsequent validation of this finding involved CT density and area measurements of thoracic and paravertebral muscles in COPD patients (42, 69, 70). Low muscle density reflects a high degree of fat infiltration in skeletal muscle, which is associated with increased disability and reduced physical function (71). Furthermore, CT measurement of the diaphragmatic crura can reflect the degree of airflow limitation in COPD patients (72). To facilitate such assessments, So Hyeon Bak's team developed an automated tool for quantifying diaphragmatic morphology on chest CT images (73), enabling easier and more efficient segmentation of the diaphragm. Nevertheless, literature on CT application in COPD remains relatively limited, presenting a promising avenue for future research.

**Figure 4:**
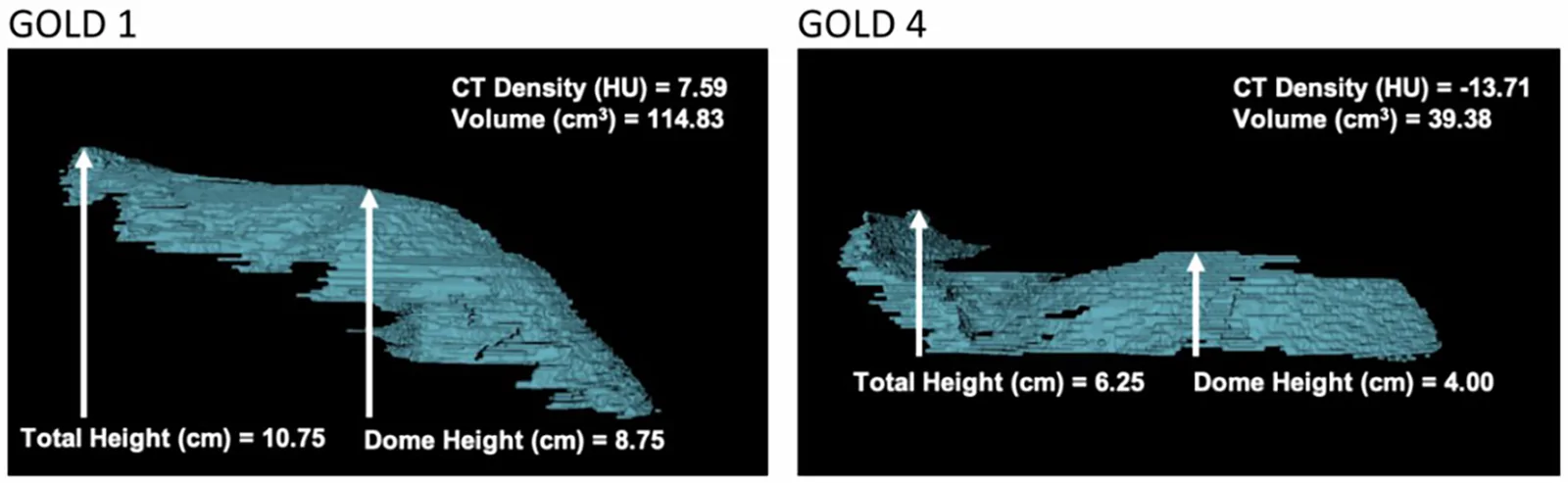
Lateral view of two representative left hemidiaphragms segmented from CT images. A threedimensional CT rendering of the diaphragm contours from segmented axial images of two representative left hemidiaphragms of mild (GOLD 1) and very severe (GOLD 4) participants with COPD. The diaphragm CT density, dome height, and volume were calculated from the three-dimensional diaphragm surface. The diaphragm dome height, measured from inferiormost axial image containing diaphragm to the central tendon region. COPD, chronic obstructive pulmonary disease; CT, computed tomography; GOLD, Global Initiative for Chronic Obstructive Lung Disease; HU, Hounsfield units. (68) 10.1513/AnnalsATS.202007-865OC.

#### 4.3.2 CT evaluation of the diaphragm in patients with VIDD

In long-term MV patients, the diaphragm experiences varying degrees of atrophy, a condition collectively referred to as ventilator-induced DD (VIDD). Several studies have indicated that VIDD is common in humans, signifying impaired diaphragmatic function, particularly among ICU patients receiving prolonged mechanical ventilation (60). Using 3D CT volumetry, Jung et al. demonstrated that diaphragmatic volume was comparable upon ICU admission but declined during hospitalization. Notably, septic MV patients exhibited a preferential pattern of diaphragmatic atrophy, with a significantly greater volume loss compared to that of the psoas (peripheral skeletal muscle); this discrepancy was absent in non-septic critically ill patients (44). In a cohort of 13 MV critically ill patients, retrospective serial clinical chest CT images (analyzed via Rapidia 2.8 3D post-processing software) were used to quantify bilateral DT at the axial celiac-axis origin level. The mean DT derived from these measurements decreased by approximately 10% during the ICU stay (51). This reduction in thickness constitutes a major factor contributing to challenges in weaning from mechanical ventilation. Yue Nan Ni et al. measured the thickness of the right hemidiaphragm dome in the coronal position, confirming that smaller losses in DT were associated with lower rates of reintubation and in-hospital mortality (74). Thus, quantifying morphological changes of the diaphragm on CT may serve as a valuable tool for assessing and guiding rehabilitation and respiratory care. Although current literature on the relationship between VIDD and CT is scarce, VIDD presents a challenging clinical problem; once developed, it can lead to difficult extubation and establish a detrimental cycle of worsening DD. Consequently, early intervention represents an effective strategy to prevent VIDD, wherein CT could play a significant role.

#### 4.3.3 CT evaluation of the diaphragm in patients with COVID-19

The COVID-19 pandemic has profoundly impacted the global population, particularly elderly patients. CT has emerged as a reliable, practical, and rapid tool for assessing the severity and prognosis of COVID-19 (75, 76). DD is recognized as a contributing factor to dyspnea in COVID-19 patients (77), with such symptoms potentially persisting for up to 15 months after hospital discharge (78). Selçuk Parlak et al. found that patients with a thinner diaphragm on initial CT scans experienced worse clinical outcomes (79). Lee et al. measured DT in MV patients at two time points, with an average interval of 18 days, observing a significant decrease in DT during mechanical ventilation. They concluded that diaphragmatic weakness is clinically relevant, particularly in pulmonary diseases where respiratory load is increased due to infections (51). Additionally, Mercan et al. calculated DT at the dome level using CT and noted that in elderly patients, a greater right-sided DT paradoxically correlated with a higher probability of ICU admission (80). This finding challenges conventional expectations, and the authors speculated that an increase in DT might not necessarily reflect better function, potentially relating to the active disease phase and angiotensin-converting enzyme receptor expression, though further validation is necessary. In a study involving 404 COVID-19 patients, Parlak et al. reported that DT measured at the abdominal aorta level was inversely associated with poor prognosis (79). Hadda et al. performed daily DT measurements in non-intubated COVID-19 patients during the first five days of admission and observed a transient decline in DT (81). Overall, CT-based DT in COVID-19 patients tends to decrease, and this reduction correlates with DD, a significant contributor to respiratory failure in these patients. While most CT studies measure DT at the level of the celiac trunk near the diaphragmatic crura, some have also utilized measurements at the dome level (59, 82). Gatti et al. conducted a study measuring DT at both the dome and crural levels using CT and subsequently investigated the correlation between these CT measurements and ultrasound assessments.

### 4.4 Disadvantages of CT

Although CT currently provides diagnostic indices and threshold values with high accuracy for assessing diaphragmatic function, it has inherent limitations, including radiation exposure, high costs, and limited applicability in patients with impaired mobility. Nevertheless, in the current healthcare context—with a sharp increase in the number of patients with pulmonary nodules in recent years, even among younger populations—chest CT has become a routine examination for hospitalized patients. Consequently, it does not impose unnecessary radiation exposure or additional financial burdens on patients. Moreover, due to its wide availability and comprehensive pulmonary imaging capabilities, chest CT is systematically employed to assess DD while providing information on other structural abnormalities beyond the diaphragm.

Overall, CT demonstrates exceptional value in diagnosing DD, particularly in patients with concomitant respiratory diseases. However, its clinical adoption is not yet widespread. Future enhancements may be achieved by integrating CT with ultrasound or phrenic nerve stimulation, aiming to better identify patients with DD and reduce the incidence of adverse clinical events.

## 5 A comparison of CT and ultrasound

First, fundamental differences between ultrasound and CT are evident in their functional capabilities. Ultrasound offers real-time imaging, allowing for direct observation of diaphragmatic movement (e.g., excursion) during spontaneous or mechanical ventilation, as well as instantaneous changes in thickness during contraction (thickening fraction). This “functional visualization” renders ultrasound the preferred tool for rapid bedside screening and dynamic monitoring of diaphragmatic fatigue and paralysis. However, significant limitations include susceptibility to interference from pulmonary gas, obesity, and right-sided anatomical structures, with approximately 10% of patients failing to obtain satisfactory images (83). Furthermore, measurements are typically performed at a single site—the right hemidiaphragm at the mid-axillary line (60)—and results are highly operator-dependent. There is also no unified consensus on the lower limit of normal values, the affected side(s) (unilateral or bilateral), or grading criteria both domestically and internationally.

In contrast, CT provides high-resolution static anatomical images that are unaffected by air-containing organs, enabling comprehensive visualization of the diaphragmatic dome, crura, and adjacent structures. Through cross-sectional scanning and 3D reconstruction, CT can reproduce the stereoscopic contour of the diaphragm and directly measure thickness and height at specific anatomical landmarks. Although CT does not display real-time contractile activity, substantial evidence supports the correlation between changes in DT and dome position with functional decline. Compared to ultrasound, CT offers superior structural delineation, objective quantitative assessments, and reveals associated pulmonary and mediastinal pathologies (84, 85). In other words, while CT infers functional status indirectly through “structural integrity,” it complements ultrasound's “direct functional observation.” Ultrasound is simpler and faster to perform than CT scanning; in certain situations—such as exploring the impact of diaphragmatic heterogeneity on patient prognosis or evaluating therapeutic strategies on different diaphragmatic regions—ultrasound may be the preferred modality. The major similarities and differences between CT and ultrasound are summarized in Table 2.

**Table 2:** Comparison of ultrasound and CT for diaphragm assessment.

Feature	Ultrasound	CT
Imaging type	Dynamic	Static
Bedside availability	Excellent	Limited
Radiation	No	Yes
Cost	Low	Higher
Operator dependence	High	Low
Diaphragm excursion	✓	×
Thickening fraction	✓	×
Diaphragm thickness	✓	✓
Diaphragm density	×	✓
Dome height	×	✓
Whole diaphragm morphology	Limited	Excellent
Three-dimensional reconstruction	×	✓
AI automatic segmentation	Limited	Available
Pulmonary lesion assessment	Limited	Excellent
Perioperative evaluation	Good	Good
ICU monitoring	Excellent	Limited
Longitudinal follow-up	Excellent	Moderate

In clinical practice, ultrasound can serve as a first-line screening tool to assess whether postoperative or MV patients exhibit reduced diaphragmatic movement. When ultrasound findings are inconclusive, the acoustic window is suboptimal, or precise preoperative planning is needed, CT can play a decisive role. For example, significantly reduced movement of one hemidiaphragm on ultrasound may indicate true paralysis or be an artifact caused by compensatory hyperfunction of the contralateral side. In such cases, CT can aid in determining the presence of structural atrophy by analyzing the symmetry of bilateral crural thickness and dome height. Additionally, for patients at high risk of diaphragmatic paralysis following brachial plexus block, routine preoperative chest CT can provide baseline anatomical data; if postoperative ultrasound raises suspicion, CT images can be re-examined to compare structural changes. This review includes a summary algorithm for selecting between ultrasound and CT, as depicted in Figure 5.

**Figure 5:**
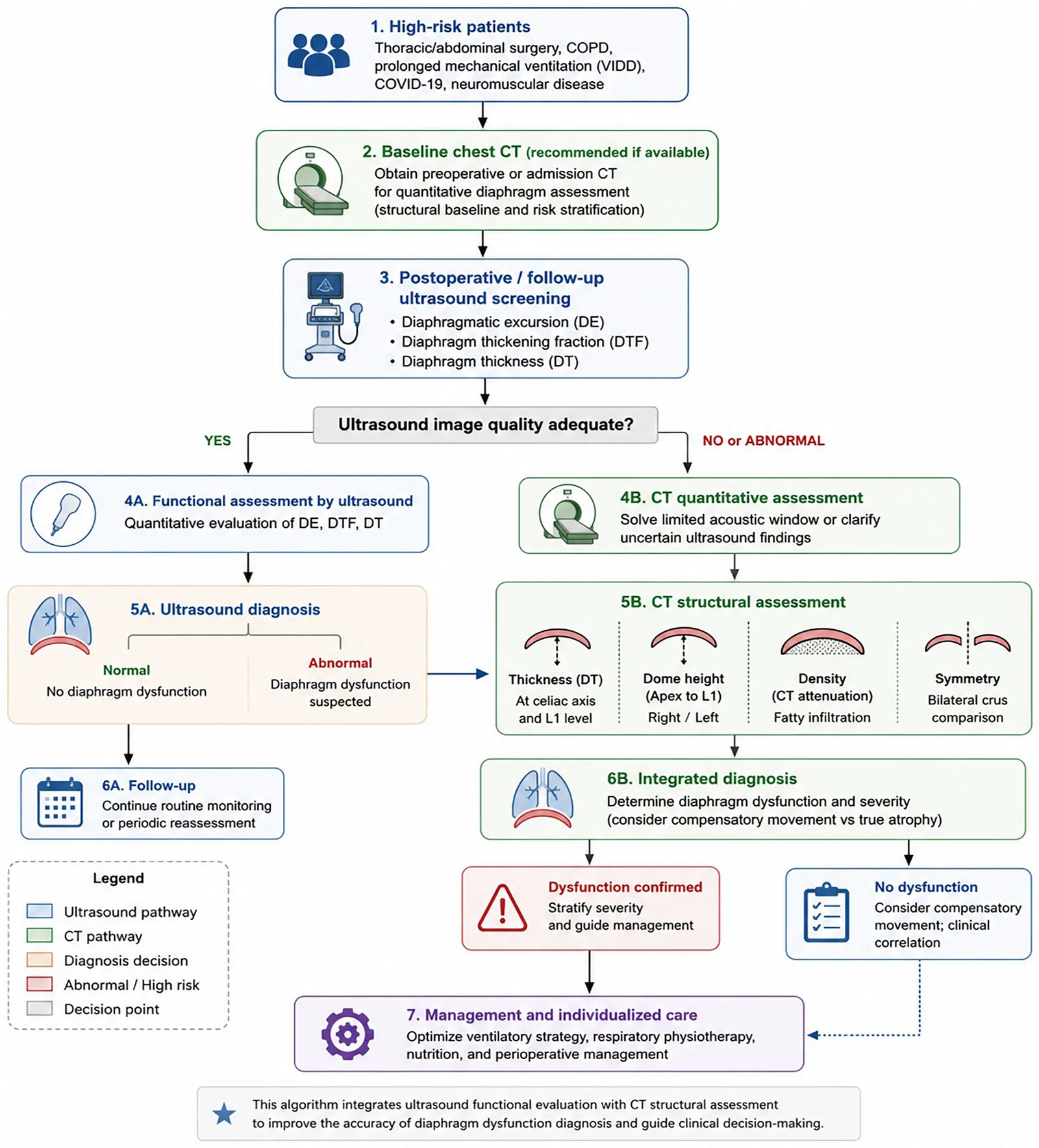
Algorithm for integrated ultrasound and CT assessment of diaphragmatic dysfunction in high-risk patients, where ultrasound acts as the primary functional screening tool and CT serves as a complementary structural evaluation method.

## 6 Discussion

Early identification of DD remains a significant clinical challenge. Timely diagnosis and intervention for perioperative-onset DD are critical to improving postoperative outcomes, particularly in the context of regional anesthetic techniques such as supraclavicular brachial plexus blockade, which carries a reported DD incidence exceeding 60%. This technique is often employed in elderly or high-risk patients for whom general anesthesia poses elevated peri-procedural risks. Therefore, practical strategies to recognize DD early and initiate appropriate supportive measures are essential.

Preoperative chest CT, routinely performed for many hospital admissions, offers high measurement reproducibility, is largely operator-independent, and enables comprehensive visualization of 3D diaphragmatic anatomy. These attributes render CT a promising modality for investigating whether baseline morphological features can stratify postoperative DD risk. Several recent studies have reported correlations between CT-derived DT and markers of diaphragmatic functional impairment. Notably, Lallement et al. proposed diagnostic cutoffs for DT measured at the abdominal aorta (3.0 mm) and at the L1 vertebral level (4.5 mm), using ultrasound-defined DD as the reference standard. It is important to emphasize that these thresholds derive from single-center data and lack external validation in independent perioperative cohorts. Although these values suggest that preoperatively reduced DT may predispose patients to clinical DD under anesthesia, mechanical ventilation, or surgical trauma, this causal or predictive effect remains unproven. Consequently, prospective multicenter studies involving heterogeneous perioperative populations are required before adopting these cutoffs for clinical risk stratification; morphological measurements alone cannot reliably replace functional assessment.

Observational data from COVID-19 and MV ICU patients further illustrate this limitation. Parlak et al. found that reduced admission DT on CT correlated with poorer clinical outcomes in patients hospitalized for COVID-19, and Lee et al. documented measurable DT loss during mechanical ventilation in critically ill cohorts. Although these associations imply that adverse clinical trajectories often occur in patients with unfavorable baseline diaphragmatic morphology, CT morphological metrics have not been established as standalone predictive biomarkers for incident DD. Moreover, CT provides global morphological descriptors unavailable via ultrasound, including bilateral dome-height symmetry, diaphragmatic muscle density, and crus cross-sectional area. Whether integrating these multiparametric CT features into preoperative risk models enhances prognostic accuracy warrants formal prospective evaluation.

Bedside ultrasound remains the primary clinical screening tool for DD. Utilizing B-mode and M-mode techniques, it captures real-time dynamic functional indices across the respiratory cycle, including DE and DTF. Advantages include the absence of ionizing radiation, good repeatability, rapid bedside deployment, and suitability for serial daily monitoring, making it indispensable for ventilator-weaning assessments in the ICU and for short-term follow-up of perioperative diaphragmatic injuries. However, several practical constraints persist: measurements are heavily operator-dependent, and suboptimal acoustic windows due to obesity, large pneumothorax, or left-sided anatomical interference can prevent adequate image acquisition in approximately 10% of patients. Additionally, a lack of international consensus on uniform diagnostic thresholds and functional grading has led to considerable inter-study variability in published normal ranges for excursion and thickness. Furthermore, ultrasound alone often fails to reliably differentiate true unilateral diaphragmatic paralysis from contralateral compensatory hyperkinesia, resulting in potential false-positive interpretations and limiting accurate risk stratification. Multi-detector spiral CT complements these weaknesses in structural assessment. Axial-coronal multi-planar reconstruction and 3D volume rendering are largely unaffected by thoracic gas and body habitus, allowing the complete visualization of bilateral diaphragmatic domes and crura. Standardized measurements of DT, dome height, and diaphragmatic muscle density enable objective quantification of muscle atrophy, fatty infiltration, and global morphological distortion. A single CT acquisition concurrently evaluates pulmonary and mediastinal pathology, aiding in the identification of secondary diaphragmatic compromise due to underlying lung diseases such as COPD, COVID-19, or intrathoracic mass lesions. Moreover, comparative analysis of bilateral crus thickness and dome height can help clarify whether reduced unilateral excursion observed on ultrasound indicates true paralysis or contralateral compensation, thereby addressing this well-documented diagnostic confounder. Nonetheless, CT provides only static anatomical snapshots and cannot directly record real-time diaphragmatic contractile behavior; functional impairment is inferred from muscle morphology rather than directly measured. Consequently, the two modalities should be regarded as complementary rather than competitive: bedside ultrasound serves for dynamic functional screening, while CT provides static structural quantification when ultrasound results are ambiguous or when baseline anatomical characterization is required.

Current literature reveals meaningful differences in CT-derived parameter patterns across patient subgroups. Heterogeneity among existing studies arises from three primary sources. First, the pathophysiological mechanisms leading to diaphragmatic injury vary by disease state. VIDD is characterized predominantly by rapid disuse-mediated atrophy, evidenced by progressive thinning at the coeliac trunk and L1 levels on CT. In COPD, chronic pulmonary hyperinflation results in overall diaphragmatic shortening, dome flattening, and fatty muscle infiltration, which manifests as reduced CT muscle density. Acute COVID-19 may cause transient diaphragmatic thickening due to systemic inflammation and tissue edema, while long-term sequelae can include secondary atrophy and decreased dome height. Injuries related to brachial plexus blocks and post-thoracic surgery typically reflect unilateral phrenic nerve damage, presenting as unilateral crus thinning and reduced dome height. These divergent injury pathways indicate that optimal CT measurement parameters are likely specific to each disease and patient population. Second, studies utilize inconsistent reference (“gold-standard”) tests for DD. Most investigations adopt ultrasound-based definitions of DD, while fewer rely on phrenic nerve electrophysiological testing or pulmonary function outcomes. Variability in the sensitivity and specificity of these reference standards contributes to the wide variation in reported CT cutoff values obtained from ROC analyses. Third, substantial baseline heterogeneity exists among study populations. Confounders such as age, BMI, nutritional state, mechanical ventilation duration, and systemic inflammatory burden have not been consistently matched across cohorts. Obesity, malnutrition, and sepsis independently drive global skeletal muscle wasting, complicating the interpretation of isolated diaphragmatic CT metrics. Collectively, these limitations highlight the necessity for disease-stratified and population-specific reference standards for CT-based diaphragmatic assessment.

## 7 Future perspectives

Current literature is notably sparse regarding perioperative patients, particularly those undergoing thoracic and abdominal surgeries. Future research directions may include: (1) establishing standardized protocols for chest CT scanning and diaphragmatic measurement; (2) validating CT-based diagnostic thresholds through multicenter prospective cohort studies; (3) constructing combined multi-parameter risk prediction models for DD; (4) developing artificial intelligence-assisted automatic segmentation tools for diaphragmatic CT; and (5) creating a stratified combined imaging diagnostic and therapeutic system with clinical validation. In the long term, as radiomics and artificial intelligence technologies mature, CT is expected to evolve from a purely anatomical morphological tool into a quantitative imaging biomarker platform capable of predicting the onset, progression, and long-term adverse prognosis of DD. However, in the short term, CT cannot replace the first-line screening role of bedside ultrasound; rather, a combined stratified assessment using both modalities represents the optimal approach for future imaging-based management of DD.

## 8 Conclusions

While ultrasound is currently the preferred bedside tool for assessing perioperative DD, it has inherent limitations, including operator dependence, restricted acoustic windows, and a lack of standardized diagnostic criteria. Conversely, CT offers high-resolution imaging, good inter-reader reproducibility, and relative insensitivity to intrathoracic gas interference. It provides promising morphological metrics, such as DT, dome height, and muscle density, which have shown observational associations with DD. CT serves primarily as a structural and complementary modality, particularly useful when ultrasound findings are inconclusive or when detailed preoperative anatomical assessment is required. Nevertheless, its capacity for definitive DD diagnosis remains unestablished, and reported diagnostic thresholds require further unified multicenter validation. While CT-derived measurements of DT, dome height, and muscle density serve as robust quantitative indicators, existing threshold values lack uniform validation, representing a promising and high-value direction for future research.
